# Repression of Smoothened by Patched-Dependent (Pro-)Vitamin D3 Secretion

**DOI:** 10.1371/journal.pbio.0040232

**Published:** 2006-07-18

**Authors:** Maarten F Bijlsma, C. Arnold Spek, Danica Zivkovic, Sandra van de Water, Farhad Rezaee, Maikel P Peppelenbosch

**Affiliations:** **1**Center for Experimental and Molecular Medicine, Academic Medical Center, University of Amsterdam, Meibergdreef, Amsterdam, Netherlands; **2**Netherlands Institute for Developmental Biology, Hubrecht Laboratory and Centre for Biomedical Genetics, Uppsalalaan, Utrecht, Netherlands; **3**Department of Biochemistry, Academic Medical Center, University of Amsterdam, Meibergdreef, Amsterdam, Netherlands; **4**Department of Cell Biology, University Medical Center Groningen, University of Groningen, Antonius Deusinglaan, Groningen, Netherlands; Stanford UniversityUnited States of America

## Abstract

The developmentally important hedgehog (Hh) pathway is activated by binding of Hh to patched (Ptch1), releasing smoothened (Smo) and the downstream transcription factor glioma associated (Gli) from inhibition. The mechanism behind Ptch1-dependent Smo inhibition remains unresolved. We now show that by mixing Ptch1-transfected and Ptch1 small interfering RNA–transfected cells with Gli reporter cells, Ptch1 is capable of non–cell autonomous repression of Smo. The magnitude of this non–cell autonomous repression of Smo activity was comparable to the fusion of Ptch1-transfected cell lines and Gli reporter cell lines, suggesting that it is the predominant mode of action. CHOD-PAP analysis of medium conditioned by Ptch1-transfected cells showed an elevated 3β-hydroxysteroid content, which we hypothesized to mediate the Smo inhibition. Indeed, the inhibition of 3β-hydroxysteroid synthesis impaired Ptch1 action on Smo, whereas adding the 3β-hydroxysteroid (pro-)vitamin D3 to the medium effectively inhibited Gli activity. Vitamin D3 bound to Smo with high affinity in a cyclopamine-sensitive manner. Treating zebrafish embryos with vitamin D3 mimicked the *smo* ^–/–^ phenotype, confirming the inhibitory action in vivo. Hh activates its signalling cascade by inhibiting Ptch1-dependent secretion of the 3β-hydroxysteroid (pro-)vitamin D3. This action not only explains the seemingly contradictory cause of Smith-Lemli-Opitz syndrome (SLOS), but also establishes Hh as a unique morphogen, because binding of Hh on one cell is capable of activating Hh-dependent signalling cascades on other cells.

## Abstract 2

The authors show that patched can inhibit smoothened activity by promoting transport of Vitamin D3 (which binds Smo), addressing a long-standing mystery in hedgehog signaling. This inhibition can occur in a non-cell-autonomous manner.

## Introduction

Signal transduction of the morphogen hedgehog (Hh) is highly unusual, with many features unique to this signalling system, many of which are only partly understood [ 1]. For instance, Hh signalling is mediated by two membrane proteins: patched (Ptch1) and smoothened (Smo). The former is a 12-pass transmembrane protein [ 2, 3] resembling the Niemann-Pick disease type C1 (NPC1) protein (which is involved in cholesterol trafficking and has a pump function); the latter is a seven-pass transmembrane protein that resembles a G protein–coupled receptor [ 4]. In the absence of the inhibitory receptor Ptch1 (or in the case of a specific mutation rendering Ptch1 dysfunctional), Smo is constitutively active and leads to the activation and nuclear translocation of its downstream transcription factor glioma associated (Gli). Under normal physiological circumstances, the activation of the signalling pathway is caused by binding of Hh to Ptch1, resulting in the internalisation of Ptch1 and, consequently, the alleviation of the inhibitory effect of Ptch1 on Smo [ 5, 6]. The exact mechanism behind Ptch1 inhibition of Smo, however, remains unclear [ 7].

**Figure 1:**
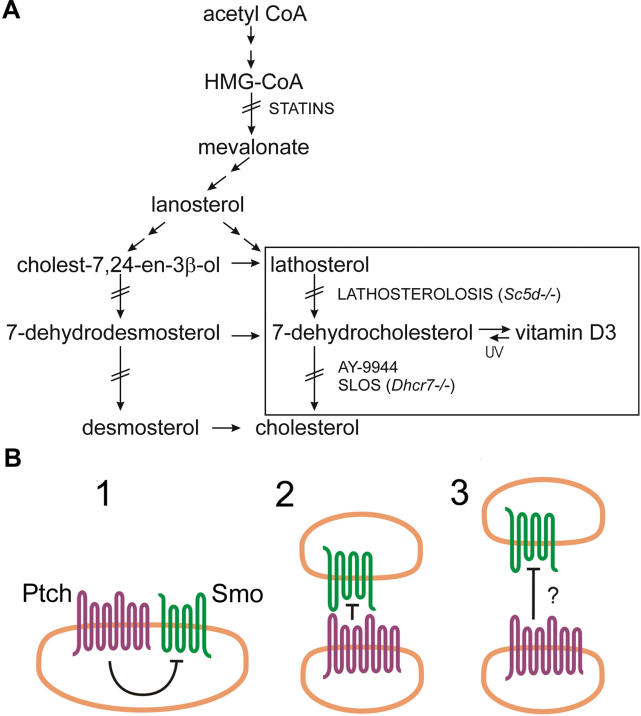
Cholesterol Synthesis and Possible Modes of Action of Ptch1 (A) Shown is a schematic representation of cholesterol synthesis. Boxed are lathosterol and 7-DHC, cholesterol precursors known to accumulate in lathosterolosis and SLOS patients, respectively. Mutations in or genetic loss of sterol C5-desaturase *(Sc5d)* cause stacking of lathosterol, whereas dysfunction of 7-DHC reductase *(Dchr7)* or the addition of its synthetic inhibitor AY-9944 causes accumulation of 7-DHC. The conversion of 7-DHC to vitamin D3 (cholecalciferol) is mediated by UV light. Statins, like pravastatin, inhibit HMG-CoA reductase, the enzyme that forms mevalonate. (B) Shown are three possible models for the inhibitory action of the Hh receptor Ptch1 on Smo. (1) A cell-autonomous mode of action, in which direct binding of Ptch1 inhibits Smo. (2) An intracellular inhibitory action, mediated by direct binding of Ptch1 to Smo. (3) The model in which Ptch1 pumps an inhibitory small molecule that is capable of Smo-repression intercellularly (as well as intracellularly; not shown).

Similarities in the phenotypes of humans with inherited disorders of sterol biosynthesis ( Figure 1A, lathosterolosis and Smith-Lemli-Opitz syndrome [SLOS]) and the phenotypes seen with mutations in the Hh signalling pathway have led to the suggestion that cholesterol-synthesizing enzymes may somehow be involved in Ptch1-dependent Smo inhibition [ 1]. Sterol content has been found to be crucial to a cell's responsiveness to Hh, independent of proper Hh sterolation [ 8], suggesting that sterol—or more specifically cholesterol—levels are pivotal to a cell's ability to respond to Hh, rather than to the accumulation of a specific inhibitory metabolite. Figure 1B depicts the proposed possible modes of Ptch1 action on Smo. Because Ptch1 and Smo do not unambiguously show physical association and because Ptch1 can inhibit Smo substoichiometrically, direct binding of Ptch1 to Smo as shown in Figures 1B, columns 1 and 2, seems an unlikely mechanism of inhibition [ 9]. Ptch1 shares high homology (for instance, a sterol-sensing domain) with NPC1, a protein involved in cholesterol trafficking [ 10], as well as with various (prokaryotic) pump proteins. Also, several small molecules with homology to cholesterol that act as Hh pathway antagonists have been identified [ 11– 13]. Cyclopamine, a well-known antagonist of Smo, has been widely used for assessing the effects of Hh pathway inhibition in several model systems (for example, in [ 14]). Consequently, it has been anticipated that in the absence of Hh, Ptch1 translocates a small molecule resembling cholesterol across the membrane, acting as a Smo antagonist. This hypothesis is summarized in Figure 1B, column 3.

A consequence of the proposed “pump” model for Ptch1 is that the action of Ptch1 should be non–cell autonomous. We set out to test this prediction, and we established that Hh activates its signalling cascade by inhibiting Ptch1-dependent secretion of the 3β-hydroxysteroid (pro-)vitamin D3. These data provide fundamental new insight into the molecular mechanisms by which Hh exerts its action in pathophysiology.

## Results

### A Model System for Measuring Ptch1-Dependent Smo Inhibition

An experimental system allowing the study of intercellular inhibitory actions of Ptch1 on Smo should fulfill four requirements: (1) cells must be capable of sustaining Ptch1 expression, (2) expressed Ptch1 must be functionally active, (3) the inhibition of Gli activity should be Smo-mediated, and (4) endogenous Hh should not be a contributing factor. To set up a model system for studying Ptch1-dependent inhibition, C3H/10T1/2 fibroblasts, which are cells extensively used for Hh research, were transfected with an 8× Gli–binding site luciferase construct [ 15] together with Ptch1, Smo, Smo and Ptch1, or Gli1.

**Figure 2:**
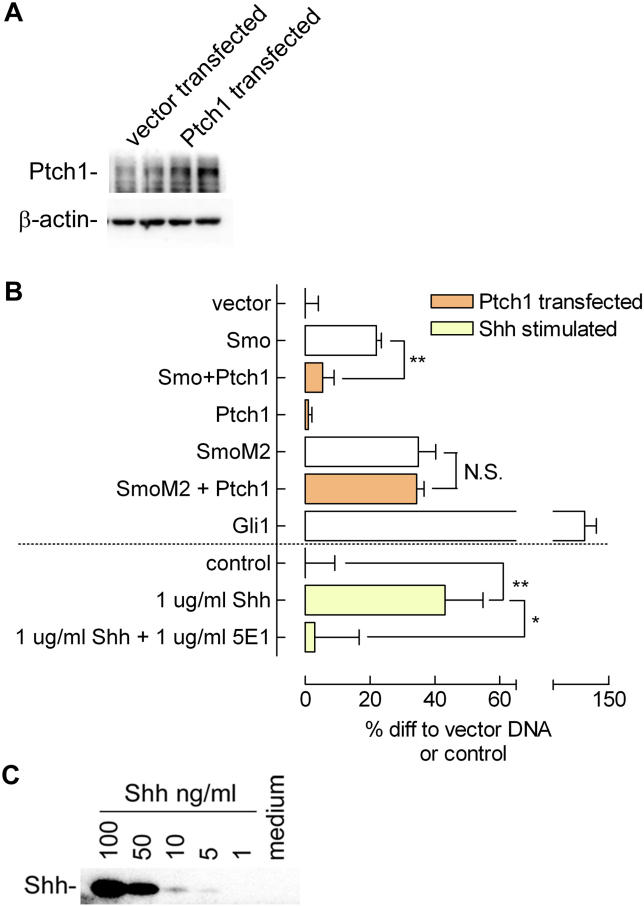
Confirmation of Functionality of Constructs and Model System Used (A) Transfection of Ptch1 expression construct in C3H/10T1/2 fibroblasts increased Ptch1 expression over basal expression, as seen on Western blot. Actin levels remained unaltered; cells were lysed 24 h post transfection. (B) C3H/10T1/2 cells are sensitive to Hh pathway components as indicated by Gli reporter activity when pathway components are expressed: Smo increased Hh pathway activity as determined by Gli reporter luciferase assay. Cotransfection of Ptch1 suppressed Smo-induced Gli activation. Transfection of Ptch1 in the absence of Smo overexpression did not decrease Gli activity below control levels. Shh stimulation (1 μg/ml for 6 h, 16 h post transfection) and transfection of a Gli1 expression construct showed highest reporter activity, as expected. Addition of 1 μg/ml Shh-blocking antibody 5E1 reduced Shh-mediated activation of Gli reporter activity. The Ptch1-insensitive mutant SmoM2 showed a high basal activity that could not be diminished by cotransfecting Ptch1, as expected. Cells were transfected and lysed after 24 h. Values shown are relative light unit (RLU) values corrected for an internal CMV Renilla standard, expressed as percent increase relative to vector (pcDNA 3.1–) transfection or control stimulation. Depicted is the mean ± SEM. ( *n* = 4; *, *p* < 0.05; **, *p* < 0.01). (C) Shh concentration in medium is below the detection limit (5 ng/ml) of Western blotting. Medium was spiked with decreasing concentrations of recombinant Shh, and blotted along with a 4× concentrated medium sample obtained from C3H/10T1/2 fibroblasts (incubated for 16 h at a volume-to-surface ratio identical to the mix-and-match and medium transfer experiments).

Overexpression of Ptch1 drove cells into apoptosis (unpublished data, consistent with [ 16]), possibly a reflection of the function of Ptch1 as a so-called dependence receptor. To overcome this problem, we performed experiments in the presence of 20 μM caspase inhibitor zVADfmk. Under these conditions, transfection with a Ptch1 expression construct led to efficient overexpression of Ptch1 as detected by Western blot ( Figure 2A). Transfection of Ptch1 effectively inhibited transactivation of the Gli reporter in the presence of overexpressed Smo ( Figure 2B). The Ptch1-insensitive mutant SmoM2 [ 11, 17] was not inhibited by Ptch1 cotransfection and showed a high basal Gli activity. Ptch1 transfection in the absence of Smo overexpression yielded no inhibition below control values, which has been described previously [ 18]. This finding suggests the presence of high basal Ptch1 levels and indicates the specificity of Ptch1 inhibition acting through Smo in our system. The inhibitory effect of the high basal Ptch1 levels could be overcome by the addition of 1 μg/ml recombinant N-terminal Sonic hedgehog (Shh) for 6 h, as can be seen from the high reporter activity upon stimulation ( Figure 2B). Addition of 1 μg/ml 5E1 Shh-blocking antibody [ 19] could counteract the stimulation by Shh. Because Smo and Gli overexpression were capable of increasing transactivation of the Gli reporter despite the presence of a caspase inhibitor, our experimental system is a valid readout for Smo-mediated Gli activity.

The requirement for Smo overexpression to respond to Ptch1 inhibition and the high responsiveness to exogenously added Shh suggest that endogenous Hh production is not a major factor in our setup, although others have shown mRNA for Indian hedgehog (Ihh) and Shh in C3H/10T1/2 cells [ 20, 21]. To exclude Hh protein excretion by our C3H/10T1/2 cells as a contributing factor in our model system, we performed Western blot analysis on Shh-spiked medium and medium from fibroblast cell culture. The cells did not excrete Hh protein ( Figure 2C). As can be seen, the detection limit was about 5 ng/ml, which was not reached in the (4× concentrated) medium, indicating that the Hh concentration in the medium was less than 1.25 ng/ml, a concentration too low to evoke a response [ 22]. Reprobing the blot for Ihh did not yield a signal for the medium either.

### Ptch1 Can Inhibit Smo in a Non–Cell Autonomous Fashion

**Figure 3:**
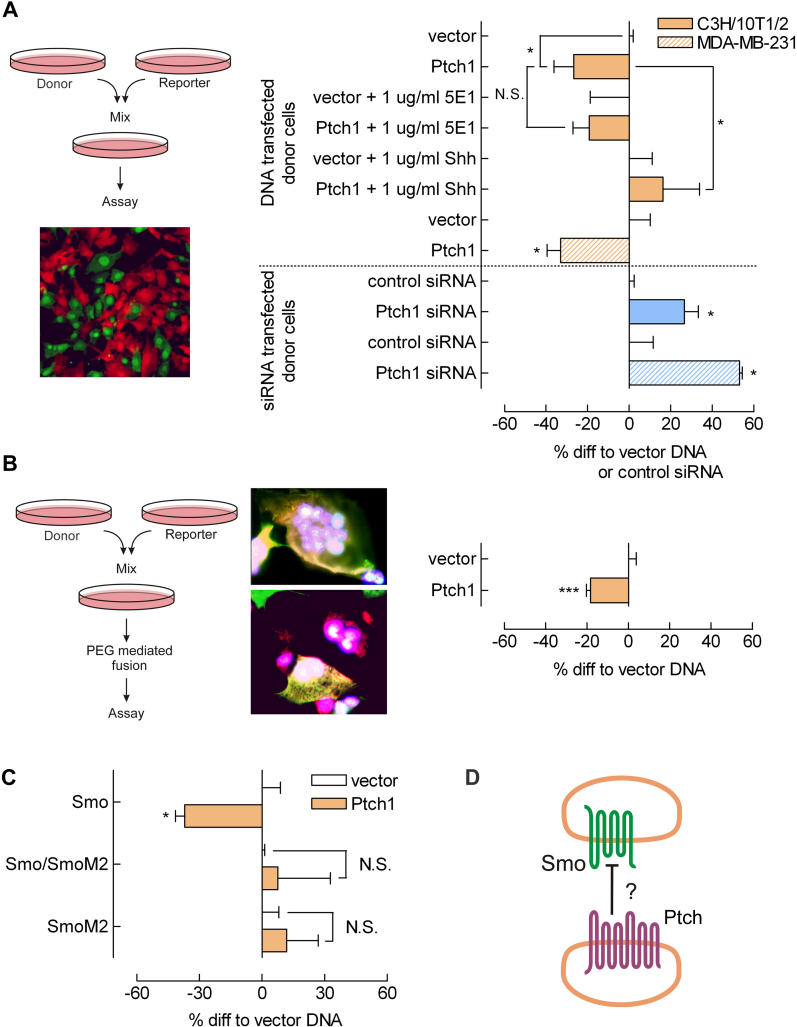
Ptch1 Inhibits Smo Intercellularly (A) Detaching and mixing of two populations of fluorescently labelled cells resulted in a homogenous mixture with intimate cell–cell contacts. The procedure is summarized in the left panel. Original magnification is 400×. Intercellular capacity of Ptch1 to inhibit Smo was assessed by mixing reporter cells overexpressing Smo and a Gli reporter with Ptch1-overexpressing donor cells. The observed decrease in Gli reporter activity could not be inhibited by the addition of 1 μg/ml 5E1 Shh-blocking antibody. Exogenously added Shh (1 μg/ml) was capable of abolishing the Ptch1-mediated inhibition. Transfecting donor cells with Ptch1 siRNA reversed the inhibitory effect (blue bars). MDA-MB-231 breast carcinoma cells (hatched bars) showed a response similar to the C3H/10T1/2 fibroblasts'. Donor cells were transfected, washed (16 h post transfection), mixed with reporter cells, and lysed after 6 to 8 h. Shown are RLU values corrected for an internal CMV Renilla luciferase control, expressed as percent differences relative to control-transfected donor cells (vector DNA or scrambled control siRNA). ( *n* ≥ 4; *, *p* < 0.05; ***, *p* < 0.005). Depicted is the mean ± SEM. (B) Fusion of reporter cells with Ptch1 overexpressing donor cells showed a decrease in Gli reporter activity comparable in magnitude to the inhibition observed in mixed cells. Right panel: treating fluorescently labelled cells with PEG1500 4 h after mixing resulted in multiple nuclei per cell and composite cell colours, indicating successful cell fusion. Magnification is 1,000×. Statistics and incubation times are as in (A). (C) To assess the specificity of Ptch1 acting on Smo, reporter cells expressing the Ptch1-insensitive mutant SmoM2 (as indicated on the *y*-axis) were mixed with donor cells. The loss of inhibition indicates the specificity of Ptch1 acting through Smo in the mix-and-match setup. Statistics and incubation times are as in (A). (D) Shown is a model for the proposed intercellular action of Ptch1 inhibition following the results obtained in the mix-and-match experiments.

To assess the ability of Ptch1 to act upon Smo on another cell, we used a “mix-and-match” approach (as schematically shown in Figure 3A), in which two populations of cells are mixed. The donor population expresses Ptch1 at different levels (using an overexpression construct or small interfering RNA [siRNA]) that could act on reporter cells with a constitutively active Hh pathway (reporter cells: Smo receptor overexpressed and a Gli-luciferase reporter). We mixed reporter cells with vector, Ptch1, Ptch1 siRNA and scrambled control siRNA transfectants. Cell populations were detached by EDTA treatment, mixed, and replated. By using two fluorescent tracers for labelling the cell populations, the capability of this procedure to obtain a homogenous mixture could be assessed. Figure 3A shows that combining two cell populations yields evenly mixed cells with intimate cell-cell contacts (micrograph). When Gli reporter cells were mixed with Ptch1- overexpressing cells in these mix-and-match experiments, a significant reduction in Smo-mediated Gli activation could be seen compared with control. This inhibition could not be diminished by addition of 1 μg/ml 5E1 Hh-blocking antibody ( Figure 3A), again demonstrating that endogenous Hh is not a contributing factor in our experimental setup. This is important, specifically because the presence of Hh in the medium would increase reporter activity. As Ptch1 scavenges Hh from the medium, medium of Ptch1-overexpressing cells would contain lower Hh levels than would control or Ptch1 siRNA-transfected cells. The reduction in reporter activity as consequence of such scavenging would mask the action of a potential inhibitory molecule. The addition of 1 μg/ml recombinant Shh, which should inhibit Ptch1 present in these experiments, was able to abolish the inhibition conferred by Ptch1. Mixing reporter cells with Ptch1 siRNA-transfected cells increased Smo-dependent reporter activity ( Figure 3A, blue bars), demonstrating that the inhibition is Ptch1 dependent and again suggests that C3H/10T1/2 cells have a significant basal level of Ptch1 activity. Showing opposite effects of Ptch1 siRNA versus DNA excludes any overexpression artefacts from responsibility for the observed non–cell autonomous effect. To exclude cell-specific artefacts, the procedures were also performed with MDA-MB-231 breast tumour cells, which had even higher levels of endogenous PTCH1 and SMO than the C3H/10T1/2 fibroblasts (Western blot, unpublished data). Indeed, using these cells yielded similar but more pronounced effects, as did the C3H/10T1/2 cells ( Figure 3A, hatched bars).

To determine the maximal inhibition Ptch1 confers in a cell-autonomous fashion—that is, the extent to which Ptch1 can inhibit Smo on the same cell—we mixed and fused the two previously described cell populations. PEG1500 induced efficient cell fusion, which was evident from blended staining of PEG1500-treated cells and the multinucleated cells (procedure and micrographs shown in Figure 3B, left panel). Fusion of reporter cells with Ptch1-overexpressing cells again showed a reduction in Gli activation, similar to that observed in the mix-and-match experiments ( Figure 3A). The reduction in reporter activity was of the same magnitude as that obtained in the mixing experiments. Therefore, we conclude that Ptch1 inhibition of Gli reporter activity in our system is mediated mainly intercellularly.

The specificity of the observed inhibitory action of Ptch1 acting through Smo was assessed by using the Ptch1-insensitive SmoM2 mutant, as previously shown in Figure 2B. As can be seen in Figure 3C, SmoM2-transfected reporter cells are no longer sensitive to mixing with Ptch1-overexpressing donor cells, even when cotransfected with wild-type Smo. These findings led us to suggest the model for Ptch1 action as depicted in Figure 1B, column 3, and Figure 3D, in which Ptch1 on one cell is capable of specifically inhibiting Smo on another cell, to act non–cell autonomously.

### Non–Cell Autonomous Ptch1-Dependent Smo Inhibition Is Carried Out by a Medium-Borne Factor

**Figure 4:**
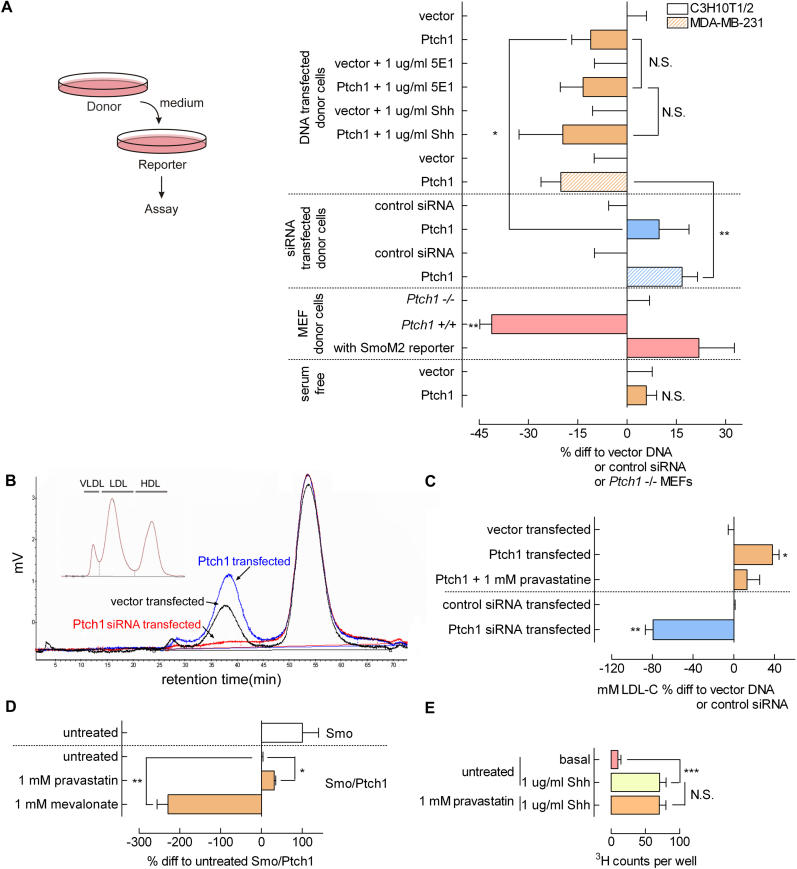
Ptch1 Secretes Smo-Inhibitory 3β-Hydroxysteroids (A) The left panel shows a schematic representation of the medium transfer experiments, in which medium was incubated on (transfected) donor cells and subsequently transferred to reporter cells. Right panel: intercellular Smo inhibition by Ptch1 is carried by the medium. Medium conditioned by control-, Ptch1-, or Ptch1 siRNA-transfected (C3H/10T1/2, solid bars, and MDA-MB-231, hatched bars) cells was transferred to Gli reporter cells. The same inhibitory action for Ptch1 was found as in the mix-and-match experiments. Neither 5E1 blocking antibody nor recombinant Shh addition to the reporter cells could diminish the inhibitory potential of the conditioned media. Medium conditioned on wild-type *(Ptch1 ^+/+^)* MEFs showed a pronounced inhibitory effect on reporter cells as compared with *Ptch1* knockout *(Ptch1 ^–/–^)* MEF-conditioned medium. Gli activity in reporter cells transfected with SmoM2 was not inhibited by *Ptch1 ^+/+^* MEF-conditioned medium. In the absence of serum, Ptch1 transfectant conditioned medium did not contain the inhibitory activity. Incubation times are as in Figure 3B. Values shown are RLU values corrected for an internal CMV Renilla standard and expressed as percent difference to control-transfected donor cells. Depicted is the mean ± SEM. ( *n* ≥ 4; *, *p* < 0.05; **, *p* < 0.01; medium Ptch1-transfected compared with medium Ptch1 siRNA-transfected). (B) Vector, Ptch1, and Ptch1 siRNA transfectant-conditioned media investigated with FPLC-coupled CHOD-PAP analysis shows loading of 3β-hydroxysteroids on lipoproteins by control cells. Medium conditioned by Ptch1-transfected cells shows a strong increase in 3β-hydroxysteroids, mainly in the LDL fraction. Ptch1 siRNA transfection abolishes 3β-hydroxysteroid loading on LDL. The inset shows the lipid standard FCS containing VLDL, LDL, and HDL. Medium was incubated on cells for 16 h. Shown are typical profiles. (C) Shown is quantification of the LDL peaks, expressed as mM. LDL-C shows the Ptch1-induced increase, a reduction by HMG-CoA reductase inhibitor pravastatin treatment, and the abolition by Ptch1 siRNA transfection of 3β-hydroxysteroid loading on LDL ( *n* = 3; *, *p* < 0.05; **, *p* < 0.01). (D) Pravastatin-inhibited Ptch1 action on Smo-driven Gli reporter activity, whereas the addition of the cholesterol precursor mevalonate enhanced this inhibition. Cells were transfected, and, after 16 h, pravastatin or mevalonate was added for 6 to 8 h. Depicted is the mean ± SEM. ( *n* = 4; *, *p* < 0.05; **, *p* < 0.01). (E) Hh-induced endocytosis is not inhibited by 1 mM pravastatin ( *n* = 24; ***, *p* < 0.005). Cells were stimulated with 1 μg/ml Shh for 1 h, and preincubated with 1 mM pravastatin or control for 6 h.

The intercellular Ptch1-mediated inhibition of Smo as identified in the mix-and-match experiments may be conferred by either an inhibitory molecule secreted or translocated by Ptch1 or by diffusion of a cytosolic mediator through tight junctions. The latter possibility is less likely, because it would result in a merged fluorophore signal in the mixed cell population, which was not observed ( Figure 3A). Indeed, important support for the possibility that Ptch1 secretes a Smo inhibitory molecule into the medium was obtained from experiments in which medium conditioned on Ptch1 *-*transfected cells was transferred to reporter cells in which Gli activity was assayed (procedure schematically shown in Figure 4A, left panel).

By using this experimental setup, we found that medium conditioned on Ptch1 *-*transfected cells exerted a strong inhibitory effect on Gli activation as compared with medium conditioned on control-transfected cells ( Figure 4A). This inhibition could not be blocked by the addition of 1 μg/ml Shh or 5E1 to the reporter cells. The inability of Shh to prevent Ptch1-conditioned, medium-dependent inhibition of Smo activity is expected, because the transferred medium does not contain donor cells expressing Ptch1 on which Shh should exert its action, and the presence of the medium-borne factor in the Ptch1-conditioned medium shortcuts the function of endogenous Ptch1 in the reporter cells. Using MDA-MB-231 cells in this setup, similar results were obtained ( Figure 4A, hatched bars). As for the mix-and-match experiments, medium conditioned on Ptch1 siRNA-transfected cells increased Smo-dependent reporter activity in both cell lines. Medium conditioned on mouse embryonic fibroblasts (MEFs) from *Ptch1* knockout mice [ 23] showed a distinct deficiency in their capacity to inhibit Smo compared with medium conditioned on wild-type ( *Ptch1 ^+/+^*) MEFs ( Figure 4, pink bars). This inhibitory effect could again not be conferred to reporter cells transfected with the Ptch1-insensitive SmoM2. The opposite effects, as observed with Ptch1 DNA transfection versus either Ptch1 siRNA transfection (blue bars) or genetic Ptch1-deficient MEFs, exclude transfection artefacts on the donor cells as the potential cause of the observed effects. Overall, these findings suggest that Ptch1 transfers a molecule to the medium that inhibits Smo activity. Medium conditioned on Ptch1-overexpressing MDA-MD-231 cells could inhibit Gli activity in C3H/10T1/2 cells and vice versa (unpublished data), suggesting that the inhibitory factor is not species-specific, at least not among mammals.

Using serum-free medium, we could confer no inhibitory action of Ptch1-conditioned medium on reporter cells ( Figure 4A). Serum-free medium contains no lipoproteins, which implies an important role for lipoproteins in conveying the inhibitory signal from cell to cell. Alternatively, serum-free medium also lacks lipids, which could stress the necessity for lipids supplied in the medium for Hh responsiveness [ 8]. However, serum-free conditions for 8 h (the incubation time in our experiments) do not deplete a cell's cholesterol metabolism, arguing in favour of a role for lipoproteins in communicating the inhibitory signal.

### Ptch1-Dependent Secretion of 3β-Hydroxysteroids

The conditioned media were subjected to fast performance liquid chromatography (FPLC)-coupled cholesterol oxidase peroxidase-amidopyrine (CHOD-PAP) analysis, a technique that analyzes 3β-hydroxysteroid content. The Ptch1-conditioned medium was found to be enriched with 3β-hydroxysteroids relative to medium conditioned by control cells ( Figure 4B and 4C; quantification of FPLC profiles as mM total 3β-hydroxysteroid). Judging from the retention profile, these 3β-hydroxysteroids were present on low-density lipoprotein (LDL) particles and, to a lesser extent, on very-low-density lipoprotein (VLDL). High-density lipoprotein (HDL) 3β-hydroxysteroid levels remained constant in all conditions. Medium incubated on cells transfected with siRNA for Ptch1 was virtually devoid of LDL-associated 3β-hydroxysteroids. This correlation between modified Ptch1 levels and the accumulation of 3β-hydroxysteroid molecules in the medium suggests that Ptch1 translocates or pumps these 3β-hydroxysteroids into the medium and thereby transfers the Smo inhibitory activity.

### 3β-Hydroxysteroids Are Necessary for Ptch1-Dependent Smo Inhibition

In the presence of pravastatin, a 3-hydroxy-3-methylglutaryl coenzyme A (HMG-CoA) reductase inhibitor that abrogates the biosynthesis of the 3β-hydroxysteroid precursor mevalonate ( Figure 1A), LDL-associated 3β-hydroxysteroid (named LDL-C on axis) levels were substantially reduced ( Figure 4C), and, correspondingly, reduced Gli inhibition was observed in Ptch1-overexpressing reporter cells as shown in Figure 4D. Conversely, the 3β-hydroxysteroid precursor mevalonate strongly increased LDL-associated 3β-hydroxysteroids and Ptch1-mediated inhibition of Gli transactivation.

If the observed effects of steroid synthesis modifiers on Gli activation are Smo-specific, Hh responses mediated by Ptch1 (but not those by Smo) should remain unaffected. The endocytosis of Ptch1 is such a Smo-independent response to Hh [ 3, 4]. By using [3H]-sucrose as a tracer [ 24], we observed no effect of 1 mM pravastatin on Hh-mediated endocytosis ( Figure 4E), and thus the effects of steroid-synthesis inhibitors are Smo-specific. The above-mentioned reporter data, however, are not from medium transfer experiments, and the inhibition or stimulation of cholesterol synthesis and the concomitant effects on Gli activity do not unequivocally prove the contribution of sterol biosynthesis to the intercellular action of Ptch1.

### Using *Dhcr7 ^–/–^* and *Sc5d ^–/–^* MEFs to Identify the Inhibitory Compound

**Figure 5:**
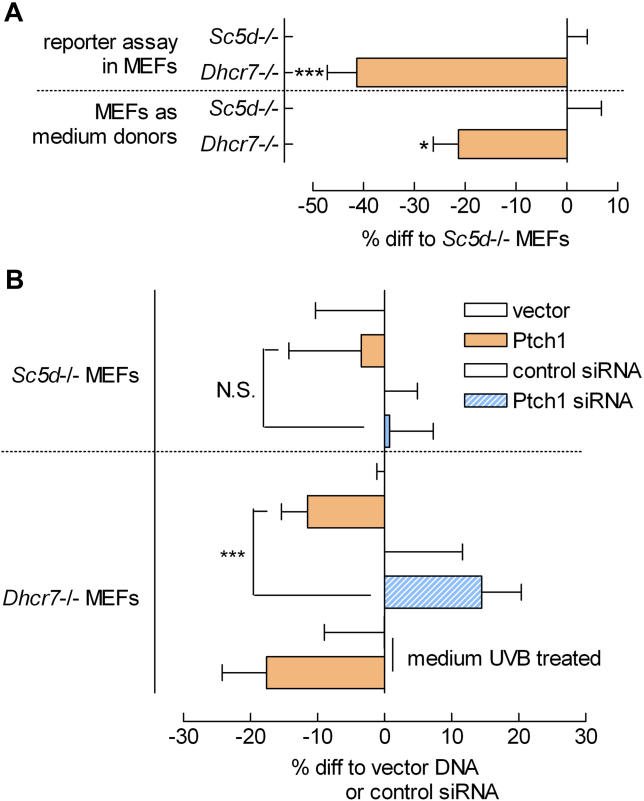
Differentially Modulated Ptch1 Action in *Dhcr7 ^–/–^* and *Sc5d ^–/–^* MEFs (A) Basal Gli reporter activity measured in MEFs either deficient for Sc5d or Dhcr7 shows that the stacking of 7-DHC (in the *Dhcr7 ^–/–^* MEFs) lowers Gli activity in the mutant cells compared with lathosterol stacking in the *Sc5d ^–/–^* MEFs. Transfer of the conditioned media to reporter cells (indicated as “MEFs as medium donors”) shows that the inhibitory potential of the *Dhcr7 ^–/–^* MEFs is medium-borne. Media were incubated on donor MEFs for 16 h and transferred to reporter cells for 6 to 8 h. ( *n* ≥ 4; *, *p* < 0.05; ***, *p* < 0.005). (B) Medium transfer of Ptch1, Ptch1 siRNA, or control-transfected *Sc5d ^–/–^* and *Dhcr7 ^–/–^* MEFs shows that cells stacking lathosterol but lacking 7-DHC ( *Sc5d ^–/–^* MEFs) are not able to translate different Ptch1 levels to an inhibitory action on reporter cells. The *Dhcr7 ^–/–^* cells were able to properly convey an inhibitory signal when transfected with Ptch1 or, inversely, show a diminished inhibitory potential upon *Ptch1* siRNA transfection. UVB treatment of Ptch1 transfectant medium (2 h) amplified the inhibitory action. Media incubations and statistics are as in (A).

The paradoxical phenotype of SLOS patients, in which diminished Hh signalling is accompanied by an accumulation of the sterol 7-dehydrocholesterol (7-DHC; see Figure 1A), led us to hypothesize that 7-DHC might be a Smo inhibitor. To test this hypothesis, we used MEFs [ 8] from mice genetically deficient for 7-DHC reductase ( *Dhcr7 ^–/–^*), because these cells stack 7-DHC. To confirm the specificity of 7-DHC as Smo inhibitor, we also used sterol C5-desaturase ( *Sc5d ^–/–^*) MEFs that stack the precursor of 7-DHC (lathosterol) and contain little or no 7-DHC. Indeed, as shown in Figure 5A, *Dhcr7 ^–/–^* MEFs had a significantly reduced Gli activity as compared with *Sc5d ^–/–^* MEFs. In addition, the Smo-inhibitory potential of *Dhcr7 ^–/–^* MEF-conditioned medium was much higher than that of *Sc5d ^–/–^* MEF-conditioned medium, as shown in the medium transfer experiment depicted in Figure 5A. In addition to stacking a specific metabolite, both MEFs are equally incapable of sterol synthesis. Our data therefore argue against reduced sterol levels as being responsible for the observed Smo inhibition. Overall, these data strongly suggest that 7-DHC or a Dhcr7-independent metabolite of 7-DHC have an inhibitory action on Smo.

To assess whether Ptch1 uses 7-DHC to inhibit Smo, we performed medium transfer experiments with Ptch1 (or Ptch1 siRNA)-transfected *Dhcr7 ^–/–^* and *Sc5d ^–/–^* MEFs as donor cells. If Ptch1 would indeed pump 7-DHC, Ptch1 overexpression or knockdown in the *Sc5d ^–/–^* MEFs should show no effect on Smo inhibition. As shown in Figure 5B, the *Sc5d ^–/–^* MEFs were severely hampered in their ability to transfer Ptch1 action to the medium, because neither Ptch1 DNA nor siRNA transfectants differed from control transfectants in their ability to inhibit Smo on reporter cells. The *Dhcr7 ^–/–^* MEFs, however, were well capable of translating Ptch1 expression levels to differential inhibitory action on reporter cells. UVB treatment of *Dhcr7 ^–/–^* MEF-conditioned media, which catalyzes the reaction from 7-DHC to vitamin D3, increased the Ptch1 effect on reporter cells, raising the tantalising option that Ptch1 uses vitamin D3 to inhibit Smo.

### Vitamin D3 Is Sufficient for Smo Inhibition

**Figure 6:**
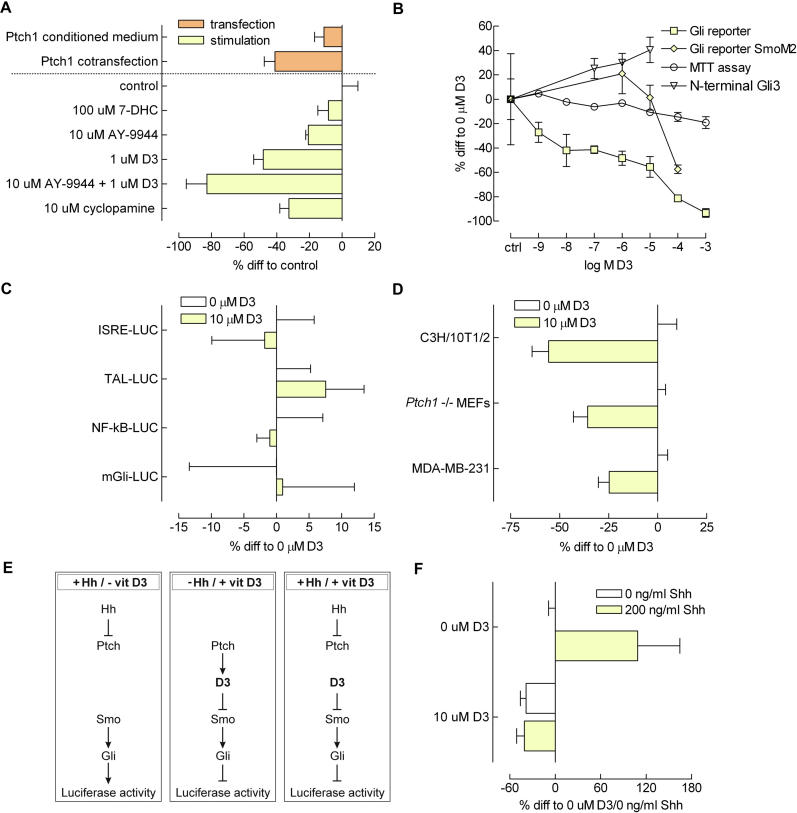
Analysis of Vitamin D3 as a Specific Smo Antagonist (A) Shown are the Gli reporter inhibitions by 7-DHC, AY-9944, a Dhcr7 inhibitor, vitamin D3, and a combination of the latter two. Also shown is the inhibition that can be conferred by the well-known Smo antagonist cyclopamine and the previously observed effects for Ptch1 transfectant conditioned medium as well as Ptch1 cotransfection (data taken from Figure 4B and Figure 2B, respectively). Cells were stimulated with the various compounds for 6 h (16 h past transfection), except for the cotransfection, which was lysed after 24 h ( *n* ≥ 4). (B) A range of concentrations of vitamin D3 was tested for inhibition of Gli reporter activity (also in SmoM2-transfected cells), MTT viability assay, and N-terminal repressor form Gli3 levels. Cells were stimulated with vitamin D3 for 6 h before lysis. For the reporter assays, RLU values were corrected for an internal CMV Renilla standard and expressed as percent difference to control stimulated cells ( *n* = 4). The MTT assays shown were raw absorption data expressed as percent difference to control stimulated ( *n* = 8); MTT agent was added for 3 h. N-terminal repressor form Gli3 was determined by quantifying ECL signal from Western blot and corrected for β-actin ( *n* = 3). Depicted is the mean ± SEM. (C) Gli specificity of the inhibitory effect of 10 μM vitamin D3 was assayed by using a panel of luciferase reporter constructs, one of which was a mGli reporter (mGli-LUC). Cells were stimulated for 6 h with vitamin D3, and the luciferase activity was assayed; values were calculated from RLU values corrected for an internal CMV Renilla standard and expressed as percent difference to control stimulated cells ( *n* ≥ 4). Depicted is the mean ± SEM. (D) Different cell types share the inhibitory response to vitamin D3 on Gli activity. C3H/10T1/2 and MDA-MD-231 cells were used earlier in the mix-and-match and medium transfer experiments. *Ptch1 ^–/–^* MEFs served as a control for any possible effects of vitamin D3 on Ptch1 function. Cells were stimulated for 6 h, and the luciferase activity was assayed,; values are RLU values corrected for an internal CMV Renilla standard and expressed as percent difference to control stimulated cells ( *n* = 4). Depicted is the mean ± SEM. (E) Proposed mechanism of vitamin D3 action. First panel: in the presence of Hh, Ptch1 is inactive and does not inhibit Smo. The Hh pathway is active, and Gli activity can be measured in a reporter assay. Second panel: in the absence of Hh, Ptch1 is active and uses vitamin D3 to inhibit Smo. The pathway is inactive, and low Gli activity is measured. Third panel: in the presence of Hh as well as exogenous D3, Smo is inhibited independently of Ptch1, and Hh can no longer elicit a Gli response. (F) Confluent Shh-LIGHT II stable Gli reporter transfectants were stimulated with 10 μM vitamin D3 and/or 200 ng/ml Shh overnight in the presence of 0.5% FCS. Luciferase activity was assayed; values are RLU values corrected for an internal CMV Renilla standard and expressed as percent difference to control stimulated cells (0 μM vitamin D3; 0 ng/ml Shh). In the presence of vitamin D3, Shh is no longer able to induce reporter activity. *n* = 4; depicted is the mean ± SEM.

From the experiments described above, we hypothesized that the addition of synthetic 7-DHC or vitamin D3 would inhibit Gli activity in reporter cells as well. Indeed, as can be seen from Figure 6A, 7-DHC was capable of inhibiting Smo, but was not nearly as potent as its derivative, vitamin D3. This fits the observation that UV treatment enhanced the inhibitory potential of Ptch1-conditioned medium ( Figure 5B). The addition of the 7-DHC reductase inhibitor AY-9944 [ 25] successfully enhanced the effect of vitamin D3 treatment but was also capable of inhibiting Smo by itself, possibly by causing accumulation of endogenously synthesized 7-DHC or by acting as a 7-DHC mimetic. The magnitude of inhibition conveyed by either the transfer of Ptch1 transfectant–conditioned medium or Ptch1 cotransfection were both smaller than that of vitamin D3. In addition, inhibition conferred by vitamin D3 was stronger than that of 10 μM cyclopamine. The finding that AY-9944 was not a necessity for inhibitory action excludes a role for sterol deprivation in this model ( Figure 1A), because the exogenously added 7-DHC or vitamin D3 can be readily metabolized by these (wild-type) fibroblasts to produce downstream sterols.

Shown in Figure 6B is a dose-dependent response of reporter cells to vitamin D3 for 6 h. In agreement, the level of inhibitory N-terminal Gli3 protein increased accordingly, as quantified from Western blot. This digestion product of Gli3 originates from proteolysis in the SuFu/Fu complex present in Hh pathway inactive cells and is considered the repressor form [ 26]. To exclude cytotoxic artefacts of vitamin D3, we measured cell viability by MTT reduction. Only at very high (1 mM) concentrations of vitamin D3 could we could observe a slight decrease in cell viability. Using SmoM2-transfected reporter cells, Gli reporter inhibition occurred only at 100 μM vitamin D3, and below that concentration, no inhibition could be observed. This is very similar to the impaired but not absent response of SmoM2 to cyclopamine [ 11].

To exclude Gli-independent effects of vitamin D3 from being responsible for the observed reporter inhibition, we used a panel of various luciferase reporter constructs. As can be seen in Figure 6C, interferon-stimulated response element (pISRE), TATA-like promoter (pTAL), nuclear factor–kappa B (pNF-κB), and mutant Gli binding site (mGli) luciferase reporter constructs were not significantly inhibited by the addition of 10 μM vitamin D3 for 6 h, whereas reporter activity of a TATA-like promoter–luciferase construct was increased. Thus, the inhibitory effect of vitamin D3 on Gli reporter activity appears to be a genuine effect of Smo-dependent signalling.

Figure 6D shows Gli reporter inhibition by vitamin D3 in the cell lines C3H/10T1/2 and MDA-MB-231 previously used in the mix-and-match and medium transfer experiments. Both showed a marked inhibition in reporter activity after treatment with 10 μM vitamin D3. *Ptch1* ^–/–^ MEFs also responded to vitamin D3. Because these cells have no Ptch1, any artefacts of vitamin D3 by interference with Ptch1 action rather than Smo activity can be excluded.

A consequence of our model in which vitamin D3 or a very similar molecule mediates Ptch1 action on Smo ( Figure 6E) is that exogenously added vitamin D3 should overrule any effect of Hh on Ptch1. To confirm this, we stimulated stable Gli-luciferase transfectants (Shh-LIGHT II) overnight with 10 μM vitamin D3 and/or 200 ng/ml Shh. What is apparent from Figure 6F is that in the presence of vitamin D3 reporter activity could not be increased by the addition of Shh, confirming our hypothesis.

### Scatchard Analysis Reveals Vitamin D3 Binding to Smo

To assess possible binding of vitamin D3 to Smo, we used a yeast strain *(Pichia pastoris)* transformed with Smo. The expression of Smo could be induced by the addition of methanol to the growth medium (kind gift of Dr. I. Mus-Veteau [ 27]). We chose this approach for two reasons: first, to our knowledge there is no vitamin D3 receptor in *P. pastoris;* second, baseline (or background) levels of Smo in noninduced P. pastoris are negligible.

**Figure 7:**
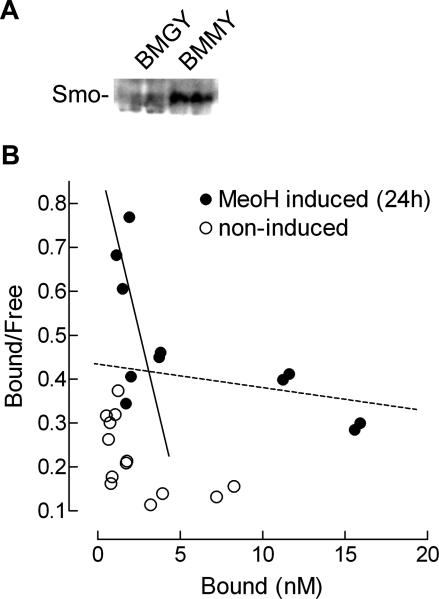
Scatchard Analysis of Vitamin D3 Using Smo-Expressing P. pastoris (A) 24 h methanol induction (buffered methanol-complex medium; BMMY) of Smo-transformed P. pastoris yielded successful Smo expression as shown by Western blot analysis with an antibody directed against Smo. (B) Scatchard analysis of heterologous competition with cyclopamine representing the binding of [3H]-D3 to Smo-expressing (solid circles, MeOH induced) and nonexpressing (open circles, noninduced) yeast strains. The solid line indicates the induced high-affinity binding, whereas the dotted line indicates nonspecific low-affinity binding. Scatchard analysis and fitting was performed as described earlier [ 53].

First we confirmed the successful induction of Smo expression by Western blot using a Smo antibody as shown in Figure 7A. We then set out to perform a Scatchard analysis [ 24] with heterologous competition by cyclopamine. Cyclopamine is a ligand for Smo, and this enabled us to distinguish the observed competition from any background by nonspecific binding or specific binding to a vitamin D3 receptor (although it is not known to exist in these cells). As can be seen from Figure 7B, [3H]-D3 was not capable of specific binding to non–methanol induced P. pastoris (BMGY), whereas after methanol induction (BMMY), specific binding of [3H]-D3 was observed that was subject to competition by cyclopamine. Cyclopamine replaced [3H]-D3 with an apparent dissociation constant *K*_d_ of 2 nM in this heterologous assay, a higher affinity than the earlier reported 20-nM affinity of cyclopamine [ 28]. Nevertheless, these results show that vitamin D3 has the potential to bind Smo at the same site as cyclopamine, indicating that vitamin D3 is indeed a physiological ligand for Smo.

### Vitamin D3 Treatment Mimics the *smo ^–/–^* Phenotype in Zebrafish

To test the action of vitamin D3 in a developmental in vivo model system, we continuously incubated zebrafish embryos starting between 32- and 64-cell stage in 1.2 mg/ml sonicated vitamin D3. We used sonication as described for 7-DHC [ 29], but did not use a stabilizing reagent. The vitamin D3 treatment phenocopied to a great extent the *slow muscle omitted (smu)* mutant phenotype [ 30], which has been identified as the zebrafish ortholog of *smo* [ 31, 32].

**Figure 8:**
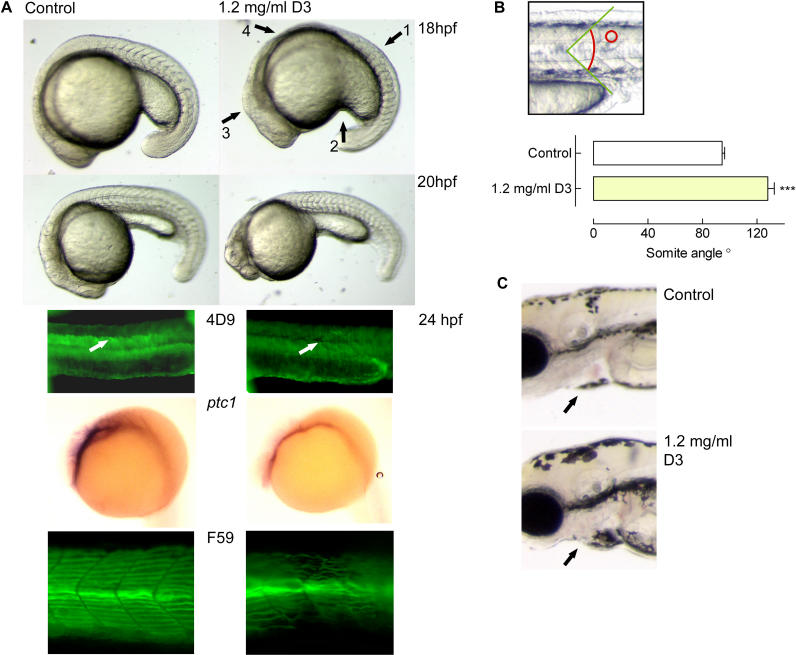
Effects of Vitamin D3 on Developing Zebrafish Embryos (A) Fertilized zebrafish eggs were dechorionated and placed in buffer containing 1.2 mg/ml sonicated vitamin D3, starting the continuous incubation between the 32- and 64-cell stages. At the different developmental stages (as indicated), the embryos were analysed, revealing persistent somite malformations, a ventrally curved body, poorly developed myoseptum, an aberrant extension of the yolk tube, a prominent dorsal midbrain, and a reduced trunk. The wild-type Engrailed staining (4D9) of muscle pioneers and surrounding cells in control-treated animals was not observed upon vitamin D3 treatment. ISH for *ptc1* mRNA showed a strongly reduced signal in vitamin D3–treated animals compared with controls. Staining with the F59 antibody revealed that slow muscle fibres were disturbed in number and orientation in the treated animals. (B) At 18 hpf, embryos were photographed in detail and somite angles were determined (as shown in top panel). The measurement was taken of an individual somite between dorsal and ventral portions of the vertical myoseptum for corresponding somites between the wild-type and vitamin D3–treated embryos. Shown are the mean angles (±SEM; *n* = 3) of wild-type control- and vitamin D3–treated embryos. C) Detail of 4-dpf embryo. Note the slightly enlarged pericardial cavity in vitamin D3–treated animals. The orientation of all images is a lateral view, anterior to the left.

One of the most striking features of the *smo ^–/–^* phenotype is the U-shaped somites in contrast to the sharply defined chevron (V)-shaped somites of wild-type embryos. The vitamin D3–treated embryos showed these disrupted somites (as indicated by arrow 1 in Figure 8A) at the developmental stages shown. Of the treated animals, approximately 75% ( *n* = 30 embryos in six individual experiments) showed this somite phenotype as well as the typical responses as listed below. Quantification of the somite angle was performed, measuring the angle between the dorsal and ventral portions of the individual somites while positioning the apex of the angle on the horizontal myoseptum ( Figure 8B, top panel). In properly shaped somites, this angle was relatively sharp, whereas in vitamin D3–treated embryos, somites were kidney-shaped, displaying a shallow angle ( Figure 8B). The ventrally curved body that is characteristic of *smo* ^–/–^ zebrafish embryos was also observed upon vitamin D3 treatment. The horizontal myoseptum was malformed ( Figure 8A; see also Figure 1 B of [ 32]). Another aspect of the *smo ^–/–^* phenotype is the aberrant extension of the yolk tube, resulting in a shorter and thicker yolk tube; this was also clearly noticeable in the vitamin D3–treated embryos (indicated by arrow 2 in Figure 8A; compare to Figure 1 A of [ 32] and Figure 4 of [ 31]). Although motoneurons have been described to be missing in *smo ^–/–^* animals [ 32], we observed no apparent difference in touch response between control and vitamin D3 animals. To our knowledge not previously reported, it appeared that the dorsal midbrain was more prominent in the treated embryos versus controls, resembling the prominent dorsal midbrain in *smo ^–/–^* embryos (indicated by arrow 3 in Figure 8A; compare to Figure 1 of [ 32] and Figure 4 of [ 31]). Also, note the similarity of the treated embryo shown at 18 h with regard to a reduced trunk, possibly caused by increased cell death as shown for *smo* mutants (indicated by arrow 4 in Figure 8A; compare to Figure 8 B of [ 33]).

To assess the specificity of vitamin D3 action on the Hh pathway, we used molecular markers specific for Hh pathway activity in zebrafish. The protein Engrailed is a marker for muscle pioneers in normal development, and as development advances, it is also expressed in the cells surrounding the muscle pioneers [ 34]. In zebrafish embryos with an abrogated Hh signalling, muscle pioneers fail to differentiate and Engrailed is totally absent [ 31, 35– 37]. No Engrailed staining of muscle pioneers (indicated by arrow in Figure 8, panel labelled 4D9) or surrounding cells was observed in vitamin D3–treated embryos, suggestive of inhibition of Hh signalling by vitamin D3. In the control-treated animals, staining of muscle pioneers as well as ventrally located cells in the somites was observed. Expression of *ptc1* as determined by in situ hybridisation (ISH) ( Figure 8A, *ptc1*) showed a marked reduction of transcript levels in the vitamin D3–treated animals. Because ptc1 is not only the primary receptor for the Hh pathway but also a transcriptional readout of Hh activity [ 32, 37], the attenuated expression seen in the treated animals confirms that vitamin D3 specifically inhibits the Hh pathway. Another marker previously described to be indicative of Hh pathway activity is slow muscle fibre orientation and number in the somites. In *smo ^–/–^* zebrafish, slow muscle fibres are fewer and malformed [ 30, 31]. Shown in Figure 8 (panel marked F59) is the disturbed pattern and number of slow muscle fibres after vitamin D3 treatment, reminiscent of *smo ^–/–^* zebrafish embryos. The use of the abovementioned molecular markers confirms that the effects of vitamin D3 on zebrafish embryo morphology are indeed due to reduced Hh pathway activity.

At later stages, we observed a subtle cardiac edema, and all treated embryos died after 5 d of vitamin D3 treatment, a feature again similar to the *smo ^–/–^* phenotype ( Figure 8C). Although we found the eyes to be relatively ventrally positioned throughout various developmental stages ( Figure 8C), no absolute cyclopia was observed. However, cyclopia is not an unambiguous hallmark of the *smo*-deficient phenotype [ 31– 33].

Because nearly all of the typical *smo ^–/–^* phenotypic features were also prominently present in vitamin D3–treated zebrafish embryos and the molecular markers used all pointed to a defective Hh signalling, we conclude that the in vivo action of vitamin D3 on embryonic development is due to a specific inhibitory effect on Smo.

## Discussion

In the present study, we investigated the molecular mode by which Ptch1 represses Smo activity. By mixing Ptch1-expressing cells with cells expressing Smo and a Gli reporter, we were able to show that Ptch1 is capable of non–cell autonomous repression of Smo. This non–cell autonomous repression is carried by the extracellular medium, because medium conditioned by Ptch1-transfected cells was capable of inhibiting Gli reporter activity as well. Conversely, medium conditioned by cells transfected with Ptch1 siRNA (so lacking endogenous Ptch1 expression) elevated Gli reporter activity significantly above medium that was conditioned by control cells, demonstrating that Ptch1 overexpression is not a prerequisite for observing this cell non-autonomous repression of Smo by Ptch1. Mixing Ptch1-overexpression cells with cells expressing Smo and Gli reporter produced inhibition of Gli reporter activity to approximately the same extent as fusing these cells, showing that this non–cell autonomous repression of Smo by Ptch1 is of roughly similar potency compared to intracellular Smo repression by Ptch1. This result suggests that this intercellular repression constitutes an important physiological mechanism. In this context, it is important to note that Hh quenching was not a major contributing factor in our experimental system, because Hh-neutralising antibodies do not interfere with Ptch1-dependent intercellular repression of Smo and we were not able to detect Hh release by the cells.

Conditioning medium with Ptch1-transfected cells led to the appearance of a 3β-hydroxysteroid in this medium, suggesting that this compound is increasingly translocated into the medium as a consequence of elevated Ptch1 expression. These results are concordant with the observation that Smo antagonists bear structural similarities to steroids [ 12] and the high homology of Ptch1 to the NPC1 protein, which has cholesterol trafficking and pump functions [ 10], as well as to various prokaryotic pump proteins. These homologies gave rise to the suggestion that, in the absence of Hh, Ptch1 pumps a compound out of the cell that binds to and inhibits Smo, and the data from this study confirm this model. Transfection of Ptch1 siRNA abolished deposition of this 3β-hydroxysteroid into the medium when compared to control-transfected cells, showing that 3β-hydroxysteroid translocation is not an artefact of Ptch1 overexpression, but that endogenous Ptch1 expression levels are sufficient to sustain detectable 3β-hydroxysteroid translocation. This 3β-hydroxysteroid translocation seems important for the intercellular repression of Smo by Ptch1, because in the presence of 3β-hydroxysteroid synthesis inhibitors Ptch1 was no longer capable of repressing Smo, whereas artificial stimulation of 3β-hydroxysteroid biosynthesis by using mevalonate supplementation enhanced the inhibitory potential of Ptch1 on Smo.

The 3β-hydroxysteroid responsible for intercellular repression of Smo is most likely the (pro-)vitamin D3 (that is, either 7-DHC or its metabolite vitamin D3) or a highly similar molecule. Fibroblasts derived from animals genetically incapable of synthesizing these 3β-hydroxysteroids *(Sc5d ^–/–^)* do not sustain intercellular repression of Smo by Ptch1, whereas fibroblasts that have a genetic defect leading to increased (pro-)vitamin D3 production *(Dhcr7 ^–/–^)* show exaggerated intercellular repression of Smo by Ptch1. Exogenous addition of vitamin D3 was more efficient in inhibiting Gli reporter activity than the established Smo antagonist cyclopamine. SmoM2, which has a reduced sensitivity towards cyclopamine, showed diminished sensitivity to vitamin D3 as well. In Scatchard assays, cyclopamine and vitamin D3 competed for Smo binding, suggesting that both compounds have an identical binding site on Smo. Scatchard analysis using the precursor 7-DHC as a competitor revealed a 10-fold lower affinity for Smo than did vitamin D3 or the hydroxylated (active) form of vitamin D3 (unpublished data). Treatment of Danio rerio embryos with vitamin D3 phenocopied most of the aspects of the *smo ^–/–^* phenotype in zebrafish. These findings strongly suggest that Ptch1 translocates either vitamin D3, a related molecule, or possibly a precursor that is subsequently metabolised in the extracellular medium that acts as a Smo antagonist.

Our findings shed light on the puzzling biochemical basis of SLOS, in which accumulation of 7-DHC ( Figure 1A) accompanies a reduced cholesterol content and a seemingly decreased Hh signalling [ 38]. Previously, the SLOS phenotype has been mimicked by using AY-9944 [ 25], which inhibits 7-DHC reductase ( Figure 1A), leading to the accumulation of its substrate. This result suggests that this molecule mediates Hh inhibition. Treatment of patients with a cholesterol-rich diet, however, has been successful, and statin treatment does not seem to alleviate the symptoms, suggesting that the lack of cholesterol is the culprit [ 38, 39]. Others have been able to counteract the effect of 7-DHC stacking in experimental settings by exogenously adding cholesterol [ 40], confirming that AY-9944 merely inhibits synthesis of cholesterol needed for proper Hh processing. Although these findings seem to preclude the stacking of a specific metabolite to be responsible for the SLOS phenotype, it has become clear that the clinical phenotype correlates best with the ratio of 7-DHC to cholesterol levels in patient plasma rather than the levels of cholesterol alone. Also, the treatment of infants with cholesterol is arguably too late an intervention, because most of the Hh patterning events are completed in utero. Our experiments show that even in the absence of AY-9944, addition of vitamin D3 strongly inhibits Hh signalling. This inhibition is stronger than Ptch1 cotransfection and Ptch1-transfectant conditioned medium, and we therefore conclude that the stacking of 7-DHC is responsible for the SLOS phenotype.

Very relevant to this manuscript and the abovementioned studies are the findings of Cooper et al., who found that reduced sterol levels impair a cell's responsiveness to Hh [ 8]. By using fibroblasts from mice genetically deficient for the enzymes involved in lathosterolosis (Sc5d) and SLOS (Dhcr7) and by depleting lipids and sterols from the medium, they showed that sterol levels were crucial to a cell's responsiveness to Hh. By analyzing the Hh protein in these cells under the various conditions, they could demonstrate proper Hh maturation, thereby excluding improper Hh sterolation as the cause of SLOS. They did not, however, hypothesize an inhibitory role for excess cholesterol precursors. We explain our hypothesis and findings in light of their results: by genetic and/or medium condition causes, steroid synthesis is seriously impaired in cells in their experiments. As a result, 7-DHC levels will drop, leaving Ptch1 devoid of substrate. Without substrate to pump or translocate, Ptch1 is “paralysed”, and addition of Hh will not exert an effect, explaining the reduced responsiveness. Hh responsiveness does not equal pathway activity, and the absence of Ptch1 substrate will diminish responsiveness but not pathway activity. Because both cell lines share the inability to properly synthesize cholesterol, and because we find a differential inhibitory potential on Smo, we conclude that sterol content alone cannot account for Ptch1-mediated Smo inhibition, and we suggest that the effects found by other groups take place elsewhere in the Hh signalling pathway. In our system, LDL seems to be a prerequisite for the diffusion of the inhibitory molecule, and, recently, a similar requirement has also been found for the distribution of Hh itself [ 41].

Although the present study clearly demonstrates that Ptch1 can inhibit Smo in an intercellular fashion, we have not addressed whether such intercellular action of Ptch1 on Smo is relevant for Hh action in vivo. Vitamin D3 is highly hydrophobic and is not expected to diffuse very far in the aqueous environment that surrounds cells. We observed that in the absence of LDL as a carrier for the hydrophobic Smo inhibitor, intercellular repression of Smo activity was undetectable. Hence, although Ptch1 exports (pro-)vitamin D3, it might act on Smo on the same cell (and thus function cell autonomously) or, depending on the cell's surroundings, on Smo on adjacent cells (and thus function non–cell autonomously). Nevertheless, the expression of Ptch1 and Smo is often discordant, with Ptch1 expression being in the vicinity of Smo-expressing cells, without complete colocalisation [ 42, 43]. We observed that in cardiac nerve branch ganglia, Smo and Ptch1 colocalise in the bronchial cartilage and epithelium, but not in the ganglion itself. In the ganglion, a pronounced staining for Ptch1 can be seen, whereas Smo staining is observed in only the adjacent cells. These findings are suggestive for intercellular signalling actually occurring in vivo (MF Bijlsma, unpublished data). In contrast with these findings are earlier reported experiments by Briscoe et al. [ 44] in which a Hh uninhabitable form of Ptch1 (Ptch1 ^Δloop2^) was evaluated with respect to its effects on Hh signalling in Drosophila melanogaster and Gallus gallus embryogenesis. The results obtained in this study suggest that the border of Ptch1 ^Δloop2^ expression more or less corresponds with the boundary of Hh-induced gene expression. Thus, it would appear that in these experimental systems, a Smo-inhibitory, vitamin D3–like ligand has not much capacity to diffuse over multiple cell layers. We therefore suggest that the action of Ptch1 can be cell autonomous or non–cell autonomous in various in vivo situations, perhaps dependent on the presence or absence of molecules that can carry the hydrophobic ligand over larger distances and subsequently present this molecule to Smo.

Recently it was demonstrated that Hh-bound Ptch1 could titrate the inhibitory action of the abovementioned Ptch1 ^Δloop2^. Furthermore, Hh-bound Ptch1 allowed Smo signalling to occur even in the presence of a Ptch1 form that is incapable of binding Hh [ 45]. This observation is inconsistent with a model in which Ptch1 serves to translocate a Smo-inhibitory molecule out of the cell, because in this model, Ptch1 action would depend on the amount of unliganded Ptch1, not the amount of liganded Ptch1. Casali and Struhl provide as a possible explanation for the titration effect that liganded and unliganded Ptch1 compete for access to Smo: Ptch1 acts as a multimer in which binding of Hh to any of the subunits blocks the action of the complex, or liganded and unliganded Ptch1 exert counteracting catalytic activities. Only the latter explanation seems consistent with the data presented in this study; the other explanation requires Ptch1 to function in a cell-autonomous fashion. Hence, it is possible that unliganded Ptch1 translocates a Smo inhibitor to extracellular medium, whereas Hh-bound Ptch1 would transport this inhibitor to the cytosol. Alternatively, Hh-bound Ptch1 is internalized, and it is possible that in this process it cointernalizes the Ptch1 ^Δloop2^ protein, especially if both proteins would localize to the same plasma membrane compartment—for example, lipid rafts. Support for this notion might be deduced from the strong Hh-dependent endocytosis we observed in the present study, suggesting that endocytosis is indeed a major cellular response to Hh binding to Ptch1. But further studies are needed to address the exact mode by which wild-type Ptch1 impairs Ptch1 ^Δloop2^ function.

Activation of the Hh pathway is, under certain conditions, associated with the development of cancer, especially in the upper digestive tract [ 46, 47] and in basal cell carcinoma in the skin, where this disease is linked to mutations in Ptch1 [ 48– 51]. Intercellular action of Ptch1 will allow this molecule to exert its action as a tumour suppressor, on not only the Ptch1-expressing cell but also neighbouring cells that may have acquired inactivating mutations of Ptch1, making this mode of Ptch1-dependent Smo inhibition especially attractive with respect to tumour suppression. Thus, it will be interesting to investigate whether intercellular repression of Smo by Ptch1 actually contributes to tumour suppression in vivo. Experiments addressing this possibility are currently in progress.

## Materials and Methods

### Constructs/siRNA used.

Mouse Ptch1 (a generous gift of Dr. M. P. Scott) was cloned into the pcDNA 3.1(–) vector (Invitrogen, Carlsbad, California, United States). Human SMO (referred to as Smo) cDNA (Image Consortium/RZPD, Berlin, Germany) was cloned into the pcDNA 3.1(+) vector. The Gli1 cDNA is in pcDNA1 (a generous gift of Dr. A. Ruiz i Altaba). The Gli-reporter δ51-LucII was kindly provided by Dr. H. Sasaki, as was the mutant binding site variant (mGli). The NF-κB, ISRE, and TAL luciferase constructs were from Clontech Laboratories (Mountain View, California, United States). The internal control cytomegalovirus (CMV) promoter-driven Renilla luciferase vector was from Promega (Madison, Wisconsin, United States). SmoM2 in pRK7 was obtained from Genentech (South San Francisco, California, United States). The Ptch1 (#63908) and scrambled control siRNA were from Ambion (Austin, Texas, United States).

### Western blotting.

Transfected cells were lysed in Laemmli buffer and brought onto SDS-PAGE gels. After electrophoresis, protein was transferred onto Immobilon-PVDF membranes (Millipore, Billerica, Massachusetts, United States). Membranes were blocked in 5% bovine serum albumin (BSA) (Sigma-Aldrich, St Louis, Missouri, United States) in TBS/0.1% Tween-20 (TBST) for 1 h. Goat polyclonal α-Ptch1 antibody G-19, α-Smo C-17, α-Gli3 N-terminal N-19, α-Shh N-19, or α-Ihh I-19 (Santa Cruz Biotechnology, Santa Cruz, California, United States) were diluted to 1:500 in 3% BSA in TBST, and membranes were incubated overnight. Goat polyclonal α-actin I-19 (Santa Cruz Biotechnology) was diluted to 1:1000 in 3% BSA in TBST. After 1 h incubation in 1:1000 α-goat HRP-conjugated secondary antibody (DakoCytomation, Glostrup, Denmark), blots were imaged using LumiLight Plus enhanced chemiluminescence substrate (ECL) (Roche, Basel, Switzerland) on a GeneGnome chemiluminescence imager (Syngene, Cambridge, United Kingdom).

### Mix-and-match procedure.

Mouse mesenchymal fibroblasts (C3H/10T1/2 from American Type Culture Collection CCL-226, Manassas, Virginia, United States) were grown in Dulbecco's Modified Eagle Medium (DMEM) (Cambrex, East Rutherford, New Jersey, United States) containing 10% fetal calf serum (FCS) (Cambrex). Human MDA-MB-231 breast carcinoma cells (American Type Culture Collection HTB-26) were grown in L-15 Leibovitz medium (Cambrex) with 10% FCS. Cells were grown to approximately 70% and transfected with either Ptch1 or Smo and the Gli luciferase reporter in combination with a CMV-Renilla luciferase internal standard using Effectene (Qiagen, Hilden, Germany) according to routine procedures. Ptch1 and scrambled control siRNA were transfected using RNAiFect (Qiagen) following the supplied protocol. Transfected cells were treated with o-methylated 20 μM zVADfmk (Sigma-Aldrich) to neutralize the apoptotic effects of Ptch1. After 16 h, cells were washed three times with phosphate buffered saline (PBS), detached by 2 mM EDTA (Sigma-Aldrich) in PBS, resuspended in DMEM with 10% FCS, and pipetted three times through a 40-μm cell strainer (BD Biosciences, San Jose, California, United States) into a tube to allow a homogenous mixture. Equal volumes of reporter and donor cells were mixed thoroughly and subsequently transferred to 24-well plates. After 6 h (4 + 2 h for the fusion protocol), cells were lysed with passive lysis buffer as provided by Promega, and luciferase activity was assayed according to the Promega Dual-Glo Luciferase Assay System (Promega) protocol on a Lumat Berthold LB 9501 Luminometer (Berthold Technologies, Bad Wildbad, Germany). Each firefly luciferase value was corrected for its cotransfected CMV-driven Renilla luciferase standard to correct for transfection efficiency or dilution effects. Recombinant N-terminal Shh was obtained from R&D Systems (Minneapolis, Minnesota, United States) and dissolved in PBS with 0.1% BSA. The 5E1 Shh-blocking antibody was from the Developmental Hybridoma Bank (Iowa City, Iowa, United States).

### Fluorescent tracking of cells.

Cells were grown to approximately 70% confluence in DMEM containing 10% FCS and washed with PBS to remove serum. CellTracker Green CMFDA or Orange CMTMR (Molecular Probes, Eugene, Oregon, United States) were diluted to 5 μM in serum-free DMEM and added to cells for 45 min, the medium was aspirated, and fresh medium containing 10% FCS was added. Cells were detached and mixed as described above. After 16 h, cells were fixed in 3.7% formaldehyde overnight or mixed as described above and subsequently fixed. For fluorescent staining of nuclei, cells were washed with PBS/0.1% Triton X-100 (PBST) and incubated in 200 ng/ml DAPI (Roche) in PBST for 1 h. Before microscopy, cells on cover slips were washed briefly in PBS, placed upside-down on another cover slip if imaged on a Leica TCS SP II confocal laser scanning microscope (Leica Microsystems, Wetzlar, Germany), or placed on an object glass when imaged on a Zeiss Axioskop (Carl Zeiss, Oberkochen, Germany).

### Cell fusion.

After fluorescent labelling (1 h) or transfection (16 h), cells were mixed, medium was aspirated, and cells were washed with PBS. Prewarmed PEG 1500/HEPES (pH 7.4) 50% (Roche) was added to the cells for 90 s, after which the cells were washed three times with PBS. Fresh medium was added, and after 2 h, cells were either lysed with passive lysis buffer and assayed for luciferase activity or fixed for microscopy after 8 h.

### Medium transfer experiments.

Donor and reporter cells were transfected for 16 h, after which the donor cells were washed extensively to wash out any remaining transfection complexes and supplied with fresh medium for 6 h. Medium was transferred to reporter cells for 6 to 8 h, after which cells were lysed and assayed for luciferase activity (according to manufacturer's protocol). The mutant MEFs were grown in complete DMEM supplemented with 15% FCS and nonessential amino acids (Sigma).

### CHOD-PAP–coupled FPLC.

Steroid levels in the main lipoprotein classes (VLDL, LDL, and HDL) were determined using high-performance gel filtration chromatography. The system contained a PU-980 ternary pump with an LG-980–02 linear degasser, FP-920 fluorescence, and UV-975 UV/VIS detectors (Jasco, Tokyo, Japan). An extra P-50 pump (Pharmacia Biotech, Uppsala, Sweden) was used for in-line CHOD-PAP enzymatic reagent (Biomerieux, Marcy l'Etoile, France) addition at 0.1 ml/min. 60 μl from each sample was subjected to size-exclusion chromatography to determine whether there is a relationship between Ptch1 expression and medium 3β-hydroxysteroid levels using a Superose 6 HR 10/30 (Pharmacia Biotech) column with Tris-buffered saline (TBS) (pH 7.4) at a flow rate of 0.31 ml/min with in-line fluorescence and UV detection on the Jasco system with CHOD-PAP assay described above. Commercially available lipid plasma standards were used for total cholesterol pattern analysis (SKZL, Nijmegen, Netherlands).

### Endocytosis assay.

Cells on 24-well plates were grown to 70% confluence and treated with no or 1 mM pravastatin for 6 h. Subsequently, 200 nCi of [3H]-labelled sucrose (Amersham Pharmacia Biotech, Freiburg, Germany) was added per well. Cells were stimulated with either 1 μg/ml Shh or solvent control (0.1% BSA/PBS) for 1 h. After washing, cells were lysed in 1% Nonidet P-40 and the lysate was transferred to 4 ml of scintillation fluid and activity was determined on a Packard Tri-Carb scintillation counter (PerkinElmer, Wellesley, Massachusetts, United States). Values were corrected for solvent control treated cells on ice.

### Stimulation experiments.

Cells transfected with the Gli-luciferase reporter were stimulated for the indicated times and concentrations with the following: cyclopamine (Biomol, Plymouth Meeting, Pennsylvania, United States), AY-9944 (Calbiochem, San Diego, California, United States), vitamin D3, or 7-DHC (Sigma).

### MTT assay.

Cells were seeded in flat-bottom 96-well plates and treated with the indicated concentrations of vitamin D3 for 6 h. During the last 3 h, 0.5 mg/ml thiazolyl blue tetrazolium bromide (MTT) was added. After incubation, supernatant was discarded, cells were lysed in 50 μl of 40 mM HCl in isopropanol, and absorbance was measured at 570 nm in a Benchmark Plus Microplate Spectrophotometer (Bio-Rad, Hercules, California, United States).

### P. pastoris culture.

The Smo-transformed P. pastoris strain was a kind gift from Dr. I. Mus-Veteau. Culture of yeast and induction of Smo expression was performed as described [ 27].

### Scatchard analysis.

Scatchard analysis was performed on intact cells as described earlier for [ 52, 53]. After 24 h growth in methanol- or glycerol-complex medium, P. pastoris strains were washed with PBS and diluted to the same OD _600_. Subsequently, aliquots of cells were labelled for 1.5 h at 4 °C in PBS containing 1.66 nM of [3H]-labelled vitamin D3 (Amersham Pharmacia Biotech) and eight different concentrations of unlabelled cyclopamine, 7-DHC, vitamin D3, or hydroxylated vitamin D3 (Sigma-Aldrich). The reaction was stopped by washing four times with ice-cold PBS. The bound radioactivity was determined by transferring the washed pellets to 4 ml of scintillation fluid, and activity was determined on a Packard Tri-Carb scintillation counter (PerkinElmer). In each experiment, each condition was performed in duplicate. In general, Scatchard plots on intact cells show considerable nonspecific low-affinity binding of [3H]-vitamin D3. Therefore, Scatchard plots were fitted according to a one- or two-site model, as appropriate. The observed points of noninduced yeast were a satisfactory fit with a one-site (low affinity) model, whereas two affinity sites could be distinguished in the induced P. pastoris.

### Animals.

Wild-type zebrafish *(D. rerio)* were kept and bred according to standard protocols. 1- to 1.5-h post fertilisation (hpf) embryos were dechorionated following 2 mg/ml pronase treatment for 1 min followed by extensive rinsing. 1.2 mg/ml vitamin D3 was added to HEPES buffered Ringer's solution and sonicated on ice for 3 min; control buffer was also sonicated. Suspension temperatures were equilibrated and added to the embryos.

### ISH and immunofluorescence.

After treatment, embryos were fixed in 4% paraformaldehyde/PBS, and ISH was carried out as described [ 54] using a *ptc1* probe synthesized according to [ 55]. The 4D9 and F59 antibodies were obtained from the Developmental Hybridoma Bank and used as previously described [ 56].

## Supporting Information

**Accession Numbers**

The Entrez Protein ( http://www.pubmedexpress.nih.gov/entrez/query.fcgi?db=Protein) accession numbers for the genes and gene products discussed in this paper are Gli1 (BAA85004), NPC1 (AAB63373), Ptch1 (NP_032983), and Smo (AAH48091).

## Abbreviations

7-DHC: 7-dehydrocholesterol
BSA: bovine serum albumin
CHOD-PAP: cholesterol oxidase peroxidase-amidopyrine
CMV: cytomegalovirus
DMEM: Dulbecco's Modified Eagle Medium
FCS: fetal calf serum
FPLC: fast performance liquid chromatography
Gli: glioma associated
HDL: high-density lipoprotein
Hh: hedgehog
HMG-CoA: 3-hydroxy-3-methylglutaryl coenzyme A
hpf: hours post fertilisation
Ihh: Indian hedgehog
ISH: in situ hybridisation
LDL: low-density lipoprotein
MEF: mouse embryonic fibroblast
mGli: mutant Gli binding site
NPC1: Niemann-Pick disease type C1
PBS: phosphate buffered saline
Ptch1: patched
RLU: relative light unit
Shh: Sonic hedgehog
siRNA: small interfering RNA
SLOS: Smith-Lemli-Opitz syndrome
Smo: smoothened
VLDL: very-low-density lipoprotein
